# Robot-Assisted Gravity Compensation for Upper Limb Motor Rehabilitation: A Systematic Review

**DOI:** 10.3390/bioengineering13050535

**Published:** 2026-05-05

**Authors:** Rodrigo Mendez, Claudia Simon Rueda, Rui C. V. Loureiro

**Affiliations:** 1 Aspire Centre for Rehabilitation Engineering and Assistive Technology, University College London, London HA7 4LP, UK; claudia.rueda.23@ucl.ac.uk (C.S.R.); r.loureiro@ucl.ac.uk (R.C.V.L.); 2 Departamento de Electronica e Informatica, Universidad Tecnica Federico Santa Maria, Concepcion 4030000, Chile; 3 National Rehabilitation Centre, Stanford Hall Estate, Loughborough LE12 5GF, UK

**Keywords:** rehabilitation robotics, gravity compensation, upper limbs, neurological disorders

## Abstract

Neurological disorders often cause severe upper limb motor impairments that restrict independence and quality of life. Robot-assisted rehabilitation enables high-intensity, task-oriented, and quantifiable training. One key feature, gravity compensation (GC), reduces the muscular effort needed to lift the limb and supports voluntary movement by offsetting the weight of the arm. This systematic review aimed to identify the types of GC strategies used in upper limb rehabilitation robots and assess clinical evidence on their effectiveness for improving motor outcomes. A search of PubMed, Scopus, Web of Science, and IEEE Xplore (January 2005–May 2025) identified 60 eligible studies: 23 describing GC implementation and 40 reporting clinical results. GC was implemented into exoskeletons, end-effectors, and sling-suspension systems through passive mechanical designs or active, model-based, and adaptive control algorithms. However, few studies reported key technical parameters such as controller algorithms, loop frequency, or tuning procedures, and only one addressed the control system stability. Clinically, GC-assisted training improved arm movement and range of motion, with greater effects in participants with higher impairment levels. However, the functional gains were modest and not superior to conventional or other robotic therapies. Substantial heterogeneity in training protocols and participants’ demographics further limits direct comparison among GC strategies. Overall, the relative effectiveness of robot-assisted GC across devices remains unclear. Standardized reporting and more clinical trials are needed to compare GC strategies within and between different types of robots.

## 1. Introduction

Neurological disorders such as stroke, traumatic brain injury, and spinal cord injury frequently result in severe upper limb motor impairments that limit independence and reduce quality of life [1,2]. Restoring arm and hand function is a primary goal of neurorehabilitation, yet recovery is often constrained by muscle weakness, abnormal tone, and impaired motor control [3,4]. Rehabilitation must therefore enable high-intensity, task-oriented practice even when voluntary movement is limited [5,6].

Over the last two decades, rehabilitation robotics has emerged as a promising tool to enhance upper limb recovery by enabling high-intensity, precisely controlled, and quantifiable training [7,8]. Robotic devices can assist or resist movement, provide feedback, and adapt to the user’s performance, offering opportunities for personalized therapy that complements conventional physiotherapy [9]. Among these technological approaches, gravity compensation (GC) has become a fundamental component of robotic rehabilitation systems [7].

Gravity compensation refers to the mechanism by which a robotic or mechanical system counteracts the torque generated by the weight of the user’s arm, effectively reducing the effort required to move against gravity. By partial or complete gravity compensation, it is easier for patients with muscle weakness to initiate and control voluntary movements [10,11]. This reduction in gravitational load can promote motor learning by enabling repeated practice of functional movements that might otherwise be too difficult or fatiguing to perform independently [12].

From a physiological perspective, GC provides a controlled environment where motor commands can be re-engaged and refined with less compensatory muscle activation [13,14]. Therapeutically, it allows for progressive challenge; support can be gradually reduced as strength and control improve, including the integration of motor learning and neuroplasticity principles into rehabilitation [15].

A variety of robot-assisted GC strategies have been proposed, differing in how the support force is modeled and controlled. Some systems use simplified arm models with constant or position-dependent torque profiles, while others incorporate adaptive algorithms that dynamically adjust support based on patient effort or limb configuration. Active robotic devices typically implement GC through software-based control schemes, whereas passive or quasi-passive devices achieve it mechanically using springs, counterweights, or elastic bands [14,16,17,18,19].

Robotic platforms and commercially available devices for upper limb rehabilitation commonly incorporate some form of gravity compensation, either as part of their mechanical design or through active control algorithms, in both clinical and research settings [20]. Although numerous studies have employed active GC within robotic designs and interventions, the specific implementation details of this feature are often only briefly described. Consequently, the current literature does not yet clarify whether particular approaches provide superior therapeutic outcomes across or within different types of rehabilitation robots [21].

Existing reviews on rehabilitation robotics have largely focused on broader topics such as assistive control modes, training paradigms, or general outcomes after neurological injury. However, few have specifically examined the concept of robot-assisted gravity compensation as a standalone therapeutic strategy. Furthermore, the relevance of factors such as accurate biomechanical modeling of the arm, real-time adaptability of support, and the distinction between passive and active implementations has not been systematically analyzed across studies [21,22,23].

Addressing these gaps is relevant to guide both clinical practice and future technology development. Understanding whether sophisticated adaptive GC algorithms lead to superior functional recovery compared to simpler or passive systems could inform design priorities and evidence-based use of robotic devices in rehabilitation settings.

Therefore, the present systematic review aims to synthesize current knowledge on robot-assisted gravity compensation for upper limb motor rehabilitation following neurological disorders. Specifically, it addresses two research questions: (1) Which robot-assisted gravity compensation strategies have been used for upper limb rehabilitation following a neurological disorder? (2) What is the current clinical evidence on the effectiveness of these strategies in improving motor function?

By systematically mapping and analyzing the available evidence, this review seeks to complement the existing literature on rehabilitation robotics and provide a structured understanding of the state of the art in gravity compensation.

## 2. Materials and Methods

### 2.1. Search Strategy and Eligibility Criteria

To address the research questions, we conducted a literature search on the 5 June 2025 in accordance with the Preferred Reporting Items for Systematic Reviews and Meta-Analyses (PRISMA) guidelines [24]. The search was carried out across four databases, Web of Science, Scopus, PubMed, and IEEE Xplore, and was limited to English-language studies published between January 2005 and May 2025. The query included the following keywords: (“neurological injury” OR “neurological impairment*” OR “stroke” OR “brain injury” OR “spinal cord injury” OR “SCI” OR “cerebral palsy” OR “CP” OR “neurological disorder*” OR “central nervous system injury” OR “CNS injury” OR “multiple sclerosis” OR “MS”) AND (“robot*” OR “mechatronic*” OR “device*” OR “system” OR “exoskeleton” OR “exosuit” OR “exo-suit”) AND (“gravity compensation” OR “antigravity” OR “anti-gravity” OR “weight compensation” OR “weight support” OR “weight counterbalance” OR “arm support”) AND (“upper limb*” OR “upper extremit*” OR “arm*”) AND (“rehabilitation” OR “restoration” OR “recovery”).

**Figure 1:**
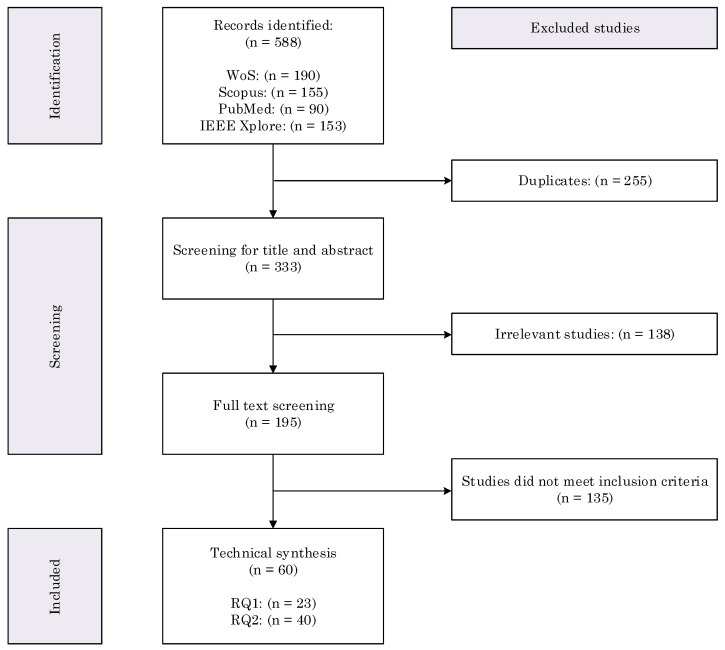
PRISMA flowchart describing the systematic process of studies identification, screening and inclusion for analysis. RQ = ’research question’.

The search led to 588 studies, reduced to 333 after duplicate removal. Two researchers (RM and CS) independently screened all records to reduce the risk of bias, any discrepancies between reviewers were resolved through discussion and consensus. Title and abstract screening narrowed the list to 195 publications, studies were considered irrelevant if they did not address gravity compensation for upper limb rehabilitation in populations with neurological disease. For full-text screening, the following inclusion criteria were applied: studies should (1) involve devices providing gravity compensation of the upper limbs, (2) focus on upper limb rehabilitation, (3) report technical details of the gravity compensation strategy (relevant to research question 1), and (4) validate the device or strategy on individuals with a neurological disorder, with biomechanical or clinical outcome measures linked to the GC intervention (relevant to research question 2). After full-text screening, 60 publications were included for technical synthesis in this review: 23 addressed research question 1, 40 addressed research question 2. Note that the total number of studies does not equal the sum of studies per research question, as some were included in both groups. Figure 1 illustrates the flowchart of the systematic search process.

### 2.2. Risk of Bias and Study Quality Assessment

The quality of the clinical trials was rated using a revised Cochrane risk of bias (RoB 2) [25] and ROBINS-I [26] for the randomized and non-randomized studies, respectively.

The quality of the rest of the studies included for clinical evidence was assessed narratively based on study design, sample size, presence of control or comparison conditions, and completeness of outcome reporting. Most included studies were feasibility, pilot, or exploratory investigations with small sample sizes.

These considerations were taken into account when interpreting the strength and generalizability of the evidence.

### 2.3. Robot-Assisted Gravity Compensation

Rehabilitation robots for the upper limbs can be classified by their mechanical configuration into three main types [27]: exoskeletons, joint-aligned frames that encapsulate the limb and apply torque at the joints; end-effectors, devices that attach to the limb at a single point; and sling-suspension systems, overhead devices that unload the limb. A fourth type has also emerged, primarily as an assistive alternative: soft exoskeletons, or exosuits, which are characterized by their construction from textile and other flexible materials [28]. Additionally, rehabilitation robots can be classified by their actuation mechanism [29]: passive (without actuators), active (with actuators), and passive–active (hybrid).

Gravity compensation can be implemented by these different robot types, accordingly, we considered both classifications when analyzing the design rationale and performance of the strategy or device, as their capabilities and operating principles are essentially different. Exoskeletons can generate a compensating torque in the elbow and shoulder independently, and end-effectors can provide support in a single point only. Additionally, active robots can implement different GC strategies, and this support usually complements a control aim [17,30]. Conversely, some devices, mostly passive, are designed solely to provide weight support and have been widely studied [31,32,33]. For this reason, the present review focuses primarily on active gravity compensation, while passive alternatives are included for comparative purposes.

To evaluate GC technical reporting of the included papers on the design and performance of the different strategies and robots, we considered and extracted the following core elements: robot classification by mechanical configuration and actuation, the arm model, the dimensions and mass considered, type of support provided (fixed or adaptive), and performance metrics (static/dynamic, muscle activation, range of motion, and other outcomes). For passive devices, we additionally analyzed the type of mechanism underpinning the device’s operating principle, while for active devices, we analyzed the specifications and performance of the control strategy.

### 2.4. Clinical Evidence

From the 40 studies included for research question 2, 34 included stroke participants (85% of the studies), 3 included multiple sclerosis (7.5% of the studies), 2 included SCI participants (5% of the studies), 2 included CP (5% of the studies), and 2 studies included Spinal Muscular Atrophy (5% of the studies). From the 34 papers that studied the effect of GC on stroke participants, 1 included participants in an acute stage, 8 in a sub-acute stage, and 30 in a chronic stage. Note that the sum of studies per stage does not equal 34, as some studies included participants in different stages.

Additionally, we stratified the participants with stroke into different groups based on pre-intervention impairment severity and/or acuity, and recorded the tests and scales used to determine their initial functioning level.

To further analyze the effectiveness of the different strategies and devices, we also assessed the study design, the training intensity based on session duration, number of sessions and frequency, and the number of participants per study.

For the synthesis of outcomes, we extracted the intervention and major findings of the clinical trials, as well as the scales or tests administered before and after the intervention. We also recorded whether follow-up assessments were performed. As per our eligibility criteria, only studies in which outcomes could be directly linked to the GC intervention were included.

## 3. Review

### 3.1. Arm Model

Regardless of whether the robot is passive or active, studies can incorporate an arm model for design purposes. Modeling the arm enables estimation of the torque or force required to compensate gravity across different arm configurations, at individual joints, and in either two- or three-dimensional space, depending on the aim of the device or GC strategy [16,19,22,34]. Active devices can additionally use this model to adjust the support provided in real time, delivering more accurate and/or adaptive assistance [17,30].

The arm is commonly modeled as two segments, each with its own mass, center-of-mass distance from the joint, and dimensions. Across the studies included in this review, three main variables are generally considered: (1) the number of degrees of freedom, (2) the dimensional representation (2D or 3D), and (3) the dimensions and weight of the arm. These choices depend on the type of robot, the aim of GC, and the simplifications adopted. With the exception of [21], all studies assumed that the center-of-mass of each arm segment lay along its longitudinal axis for simplicity.

Additionally, even when a study models the arm in 3D or the device operates in a 3D workspace, gravity compensation may still be provided only in a two-dimensional space. In such cases, assistance typically supports only the torque generated at the shoulder in a single plane, usually shoulder flexion/extension, and/or at the elbow for flexion/extension [19,35].

Among the studies included for research question 1 and for gravity compensation purposes, approximately 52% followed a 3D arm model with four or five degrees of freedom, while 48% followed a 2D model with two degrees of freedom. Furthermore, 52% of studies used anthropometric data, e.g., following [36] or [37], to estimate the dimensions and mass of the arm segments, and thus the amount of support required, while 13% obtained these values empirically using sensors with the arm fully relaxed in a defined posture and/or directly measuring the dimensions of its segments. In addition, 13% used a combination of anthropometric and empirical data, whereas this information was not reported in 22% of studies.

Following Lagrange’s formulation, the arm model can be expressed as

(1) $$M_{h}\left(q_{h}\right){\ddot{q}}_{h}+C_{h}\left(q_{h},{\dot{q}}_{h}\right)q_{h}+g_{h}\left(q_{h}\right)=\tau_{h}\;$$

where $q_{h}$, ${\dot{q}}_{h}$ and ${\ddot{q}}_{h}\in R^{n}$ are the generalized coordinates and their first and second derivatives; $M_{h}\left(q_{h}\right)\in R^{n\times n}$ is the inertia matrix; $C_{h}\left(q_{h},{\dot{q}}_{h}\right)\in R^{n\times n}$ is the Coriolis/centrifugal matrix; $g_{h}\left(q_{h}\right)\in R^{n}$ is the gravity vector; and $\tau_{h}\in R^{n}$ is the vector of joint torques generated by the human. The subscript *h* denotes human, distinguishing these variables from those of the robotic device. A friction term may also be included in the model [38].

Considering now the torque applied by the robot, we can express the model as

(2) $$M_{h}\left(q_{h}\right){\ddot{q}}_{h}+C_{h}\left(q_{h},{\dot{q}}_{h}\right)q_{h}+g_{h}\left(q_{h}\right)=\tau_{h}+\tau_{r}\;$$

where $\tau_{r}\in R^{n}$ represents the torque applied by the robot.

The goal of gravity compensation is to partially or fully counteract the gravity term $g_{h}\left(q_{h}\right)$ by applying a torque $\tau_{r}$ such that

(3) $$\tau_{r}=\Lambda g_{h}\left(q_{h}\right)\;$$

where $\Lambda \in R^{n\times n}$ is a diagonal matrix whose elements lie between 0 and 1. This goal is, however, constrained by the capabilities of the robot [16].

**Figure 2:**
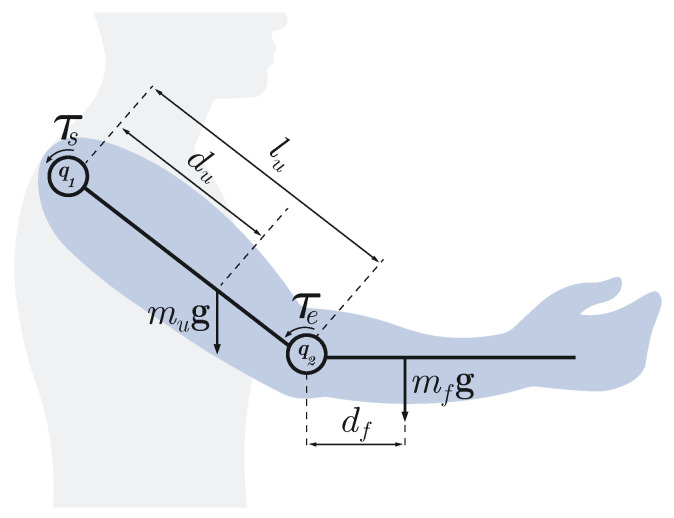
Two-dimensional representation of the arm illustrating the variables and parameters used to estimate the torques generated by the weights of the arm segments at the shoulder ($\tau_{s}$) and elbow ($\tau_{e}$). $l_{u}$ denotes the length of the upper arm; $d_{u}$ and $d_{f}$ denote the distances from the shoulder and elbow to the centers of mass of the upper arm and forearm, respectively; $q_{1}$ and $q_{2}$ represent the shoulder and elbow joint angles, respectively; $m_{u}$ and $m_{f}$ represent the masses of the upper arm and forearm, respectively; and g denotes the gravitational acceleration.

For illustrative purposes, consider the two-dimensional case depicted in Figure 2, n=2 and the gravity term is represented by a uni-dimensional torque at the shoulder $\tau_{s}$, associated with shoulder flexion/extension, and a uni-dimensional torque at the elbow $\tau_{e}$, which can be expressed as

(4) $$\tau_{s}=\tau_{su}+\tau_{sf}$$

(5) $$\tau_{e}=\tau_{ef}\;$$

where $\tau_{su}$ and $\tau_{sf}$ are the torques generated at the shoulder by the upper arm and forearm, respectively, and $\tau_{ef}$ is the torque generated at the elbow by the forearm. This representation is commonly followed because the gravity torque generated about the shoulder flexion/extension is typically the largest [22]. Furthermore, some devices or strategies are designed to compensate for this torque only [39,40], even when they operate in a three-dimensional workspace [19,35].

The magnitudes of these torques can be expressed as functions of the shoulder and elbow angles (Figure 2) as follows:

(6) $$\tau_{su}\left(q_{1}\right)=d_{u}sin\left(q_{1}\right)m_{u}g\;$$

(7) $$\tau_{sf}\left(q_{1},q_{2}\right)=\left(l_{u}sin\left(q_{1}\right)+d_{f}sin\left(q_{2}-q_{1}\right)\right)m_{f}g$$

(8) $$\tau_{ef}\left(q_{1},q_{2}\right)=d_{f}sin\left(q_{2}-q_{1}\right)m_{f}g$$

where $q_{1}$ and $q_{2}$ are the shoulder and elbow flexion/extension angles respectively; $d_{u}$ is the distance from the shoulder to the center-of-mass of the upper arm; $d_{f}$ is the distance from the elbow to the center-of-mass of the forearm (and hand), assuming the centers-of-mass lay along the longitudinal axis of each segment; $m_{u}$ and $m_{f}$ are the masses of the upper arm and forearm respectively; and *g* is the gravitational acceleration.

**Figure 3:**
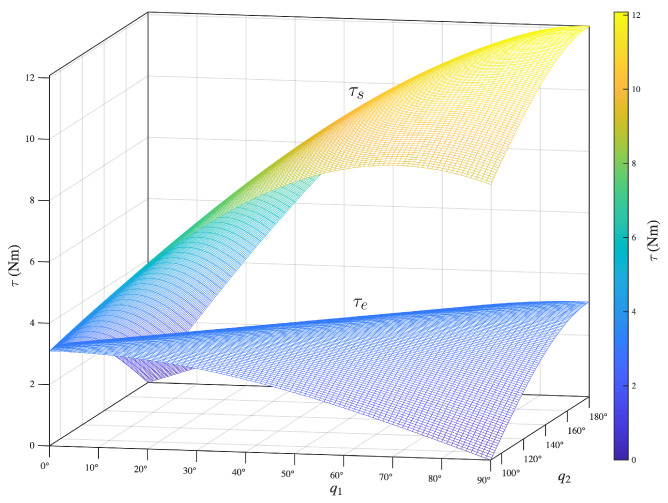
Torque magnitudes at the shoulder ($\tau_{s}$) and elbow ($\tau_{e}$) generated by the weights of the arm segments during movement in the sagittal plane, with $q_{1}\in \left[0^{\circ },90^{\circ }\right]$ and $q_{2}\in \left[90^{\circ },180^{\circ }\right]$, where $q_{1}$ and $q_{2}$ denote the shoulder and elbow joint angles, respectively. The analysis considers an adult male subject, 1.80 m tall and weighing 80 kg, with arm segment masses and dimensions estimated according to [36].

To depict how these magnitudes vary, consider an adult male subject, 1.80 m tall and weighing 80 kg. Following [36], each segment’s dimensions and mass can be estimated. Figure 3 shows $\tau_{s}$ and $\tau_{e}$ for $q_{1}\in \left[0^{\circ },90^{\circ }\right]$ and $q_{2}\in \left[90^{\circ },180^{\circ }\right]$.

This two-dimensional example illustrates: (1) how the torque magnitudes vary; (2) that the shoulder flexion/extension torque is considerably larger than the elbow torque; (3) the main parameters influencing the magnitude of these torques; (4) their dependence on arm position, showing that elbow torque can be fully compensated with an external constant force, whereas shoulder torque cannot; and (5) that simultaneous full compensation of shoulder and elbow torques is not possible with an end-effector robot. This limitation explains why some studies using end-effector devices incorporate an additional gravity-support mechanism [41,42].

This analysis can be extended to a three-dimensional representation of the arm, typically modeled with three degrees of freedom at the shoulder and one at the elbow [16,30]. Unlike the two-dimensional case, the inverse kinematics problem does not have an explicit solution; instead, recursive algorithms are required for real-time estimation of the torques or forces at different arm positions.

### 3.2. Passive Devices

Passive devices primarily consist of mechanical systems that provide static balance using counterweights, springs, cams, or elastic elements such as rubber bands. The main advantages of these devices are their low inertia, reduced complexity, and lower cost compared with active robots [7]. Of the 23 studies selected for research question 1, eight described passive devices [14,34,35,39,43,44,45,46], five described hybrid devices [17,19,47,48,49], and one described a passive and an active version of the same robot [50]. In the hybrid systems, the passive component was mainly incorporated to provide partial gravity support to reduce actuator size requirements and improve safety, for example, in case of power failure [17,47], or to provide full gravity compensation of the robot and arm [48]. In [19,49], the passive component of these hybrid robots is a gimbal used as a human–machine interface to align the robot with the natural motion of the user’s wrist or forearm.

Of the twelve studies that describe passive gravity compensation, five involve exoskeletons [17,35,44,46,50], four end-effectors [34,39,45,47], two the same sling-suspension concept [14,48], and one an orthosis [43].

In terms of gravity compensation, all devices except [43] follow widely studied mechanical design concepts. The latter describes an orthosis that supports the glenohumeral (GH) joint without restricting shoulder motion in three-dimensional space, within a considerable workspace. This is achieved through a parallelogram mechanism that applies a pair of compensating forces at the GH joint center, which counteract each other’s induced moments so that no additional moment is applied to the joint.

To assess device performance, five of the twelve studies primarily measured the range of motion and muscle EMG while participants performed dynamic movements [14,17,34,43,46]; three studies did not clearly specify the tests performed during user effort, range-of-motion or usability measurements [35,45,47]; one study evaluated static support using weights as a load [39]; and three studies did not report any evaluation related to gravity compensation [44,48,50].

### 3.3. Active Devices

Active rehabilitation robots are typically electromechanical devices whose main advantage, compared with passive ones, is their flexibility to provide different types of therapy. The assistance provided can be automatically adjusted in real time based on, for example, the user’s performance [10]. Of the 23 studies selected for research question 1, twelve describe an active gravity compensation strategy. Five of them are focused on exoskeletons [17,21,30,38,51], five on end-effector robots [16,19,22,49,52], and two on sling-suspension devices [40,53].

In active robots, upper limb gravity compensation can be either a primary or a secondary aim of the control strategy; furthermore, it may be designed to partially or fully compensate for the torques generated at the shoulder and/or elbow joints, depending on the purpose of the device and its mechanical constraints.

#### 3.3.1. Sling-Suspension Devices

Sling-suspension devices are commonly simple and cost-effective passive devices designed solely to provide gravity compensation for assistive or rehabilitation purposes [12]. Their main limitations are their ability to provide only pulling forces, ideally in a vertical plane, to avoid unwanted horizontal pulling forces.

Among the active implementations, only two actuated sling-based systems were identified [40,53,54]. Both rely on simplified biomechanical representations to estimate support torques, despite operating in three-dimensional workspaces. In the system described by Scotto et al. [40], gravity support is generated by combining a proportional–derivative tracking term with a scaled gravity torque estimated empirically, rather than from a full inverse-dynamics formulation. Similarly, the Diego system employs a two-dimensional sagittal-plane arm model, assuming proportional shoulder–elbow torques and horizontal forearm posture, even though the device allows three-dimensional motion.

Across these systems, controller structure and stability properties are either minimally described or not reported. Together, these findings indicate a consistent trade-off in sling-suspension devices: while they offer practical and scalable gravity unloading, their simplified modeling assumptions and limited validation constrain their ability to provide precise, directionally consistent support across the full upper limb workspace.

#### 3.3.2. End-Effector Devices

End-effector robots, unlike exoskeletons, are anchored to the limb at a single point, usually around the forearm or wrist. This characteristic constrains their ability to simultaneously provide full gravity compensation for the torques generated at the shoulder and elbow by the arm segment masses. As a result, gravity compensation in end-effector systems is necessarily implemented as an indirect force-matching problem, rather than as joint-level torque control.

Five studies investigated active end-effector devices for gravity compensation [16,19,22,49,52]. Across these systems, gravity support is generated indirectly, by projecting the gravitational load of the multi-joint upper limb into a single interaction force at the hand or forearm. Because an end-effector cannot directly compensate joint-level torques, all strategies rely on simplified biomechanical models and optimization or scaling schemes to approximate the desired support.

Most devices employ reduced-degree-of-freedom arm models to estimate the gravitational load. Manzano et al. [22] used a two-dimensional, single-degree-of-freedom arm representation to support shoulder flexion, while AREBO [49] estimated shoulder flexion/extension and abduction/adduction torques using a two-degree-of-freedom shoulder model without external sensors. In contrast, Zhang et al. [52] combined robot kinematics with motion capture to estimate a four-degree-of-freedom arm pose in real time, enabling three-dimensional gravity compensation. Similarly, the EMU robot [16,19] computes a sagittal-plane shoulder torque equivalent to the combined gravitational effects of the upper arm and forearm. Despite these differences in model complexity, all approaches share the same underlying limitation: proximal joint torques must be inferred from a distal contact point, introducing unavoidable approximation.

To map the estimated arm gravity to an end-effector force, the reviewed systems adopt either optimization-based or scaling-based strategies. Manzano et al. [22] compared four optimization criteria, including energy balancing and torque minimization, and found that potential energy balancing yielded the greatest effort reduction in static tests. Zhang et al. [52] and Crocher et al. [16] instead formulated gravity compensation as a force-matching problem, minimizing the norm of the difference between the human arm gravity term and the end-effector force. AREBO [49] introduced a performance-based scaling factor to adapt support according to user success, highlighting a shift toward assistance-as-needed paradigms.

Overall, these studies indicate that end-effector gravity compensation strategies trade biomechanical fidelity for mechanical simplicity and adaptability. While higher-dimensional models and optimization schemes can improve the estimation of gravitational loads, the indirect nature of end-effector support limits directional consistency and anatomical precision. This fundamental constraint may contribute to the heterogeneity observed across platforms.

#### 3.3.3. Exoskeletons

Five studies focused on active exoskeleton devices were included in this review. In contrast to end-effector systems, exoskeletons enable direct joint-level torque compensation, allowing gravity support to be computed and applied in the same coordinate space as the human arm. Consequently, all reviewed exoskeletons implement gravity compensation as a feedforward torque term derived from a biomechanical model of the upper limb, integrated into the robot’s control loop.

Across platforms, the main design variable is the fidelity of the arm model used to estimate gravitational torques. The NTUH-ARM [30,38] employs a four-degree-of-freedom dynamic arm model mapped from the exoskeleton joint angles to estimate target accelerations and corresponding torques while partially or fully compensating gravity. This model-based feedforward strategy is formally analyzed for stability in [30], which remains one of the few examples in the literature to include a Lyapunov-based stability proof of gravity compensation.

Similarly, the ARMin exoskeleton [21] explores three gravity-compensation strategies that differ solely in how the arm is represented: from simplified independent segments using anthropometric parameters [51], to empirically estimated three-dimensional segment volumes, to an empirically coupled shoulder–elbow formulation. These variants demonstrate that increasingly complex models can be integrated within the same control architecture, but they also reveal the absence of consensus on the level of anatomical detail required to achieve meaningful functional benefit.

The AGREE exoskeleton [17] further illustrates this paradigm by computing a desired gravity-support torque using a recursive Newton–Euler inverse dynamics formulation that accounts for both robot and human segment weights. A weighting factor enables partial gravity compensation, and the feedforward torque is applied at high control rates (1 kHz for gravity computation and 5 kHz for torque control).

Taken together, these studies indicate that active exoskeleton gravity compensation is conceptually consistent across platforms, relying on the inverse-dynamics feedforward control to directly counteract gravitational torques. However, despite substantial differences in model complexity and computational implementation, no study demonstrates a clear functional advantage of higher-fidelity modeling over simpler formulations. This suggests that, while exoskeletons offer superior anatomical precision compared to end-effector devices, the clinical relevance of increasingly complex gravity models remains to be established.

#### 3.3.4. Gravity Compensation Assessment

To assess device performance, four of the twelve studies implementing active GC primarily measured muscle EMG while participants performed dynamic movements [17,30,40,52]; three studies evaluated the performance of the GC only statically [16,22,51]; two studies assessed the GC strategy dynamically and statically [21,38]; and three studies did not report any evaluation related to gravity compensation [19,49,53].

### 3.4. Clinical Evidence Findings

**Figure 4:**
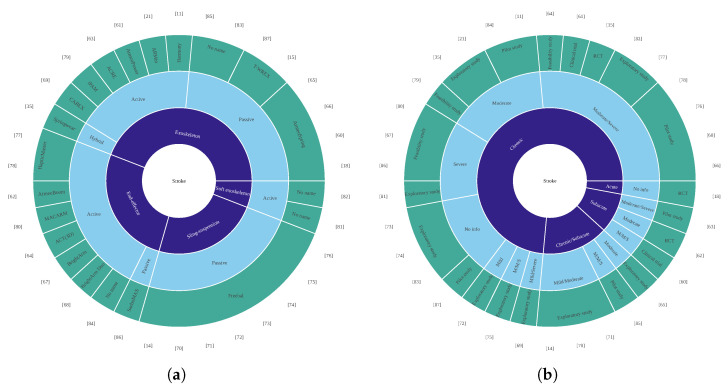
Overview of the studies included for clinical comparison assessing the device in participants affected by a stroke [11,14,15,18,21,35,60,61,62,63,64,65,66,67,68,69,70,71,72,73,74,75,76,77,78,79,80,81,82,83,84,85,86,87]. Each row represents a different study with its corresponding reference. (**a**) Robots used in each study and their classification. (**b**) Participants’ demographics and study design. RCT = randomized clinical trial; M/M/S = mild/moderate/severe. Note that multiple studies may use the same robot.

This section provides an overview of the main characteristics of the clinical evidence on gravity compensation, with further details presented for the clinical trial studies. Note that participants in the control groups were not included in the analysis. Table 1 provides an overview of the studies included in the clinical comparison that tested the devices in participants affected by neurological conditions other than stroke [42,55,56,57,58,59], Table 2 presents a more detailed summary of the clinical trials [15,18,58,60,61,62], while Figure 4 provides an overview of the studies included in the clinical comparison assessing the device in participants affected by a stroke [11,14,15,18,21,35,60,61,62,63,64,65,66,67,68,69,70,71,72,73,74,75,76,77,78,79,80,81,82,83,84,85,86,87].

#### 3.4.1. Risk of Bias and Study Quality Findings

All randomized trials showed some concerns for risk of bias, primarily related to lack of participant and therapist blinding and absence of trial preregistration, although outcome assessors were blinded and validated measures were used. All non-randomized clinical trials were judged at serious risk of bias, mainly due to confounding from concurrent conventional therapy and non-randomized designs. These limitations reflect structural challenges in rehabilitation robotics research rather than isolated methodological flaws. Table 3 and Table 4 present the risk of bias assessment for the RCT and non-RCT studies, respectively.

#### 3.4.2. Participants’ Demographics

Most of the studies included for research question 2 recruited participants with stroke (85%), the majority of whom were in the chronic stage (88.2%), followed by the sub-acute stage (23.5%) and, lastly, the acute stage (2.9%). Note that the total exceeds 100% because some studies included participants at different stages. Additionally, 7.5% of the studies included participants with multiple sclerosis, 5% included participants with SCI, 5% with CP, and 5% with spinal muscular atrophy.

#### 3.4.3. Experimental Protocol

Similar to previous reviews [13], a high degree of variability was observed in the number of participants (13.9 ± 10.4), number of sessions (4.5 ± 9.6), session frequency (times per week; 3.2 ± 1.3), and session duration (56.9 ± 35.4 min). Two studies did not report the number of sessions clearly [42,80], 17 studies (42.5%) involved a single session, 17 studies (42.5%) did not provide session-duration information, and four studies were excluded from the session-duration analysis because they reported large variability in this measure.

Of the studies included for research question 2, 45% were exploratory studies, 25% pilot studies, 12.5% feasibility studies, 7.5% clinical trials, 7.5% randomized clinical trials, and one was a single-case report.

**Figure 5:**
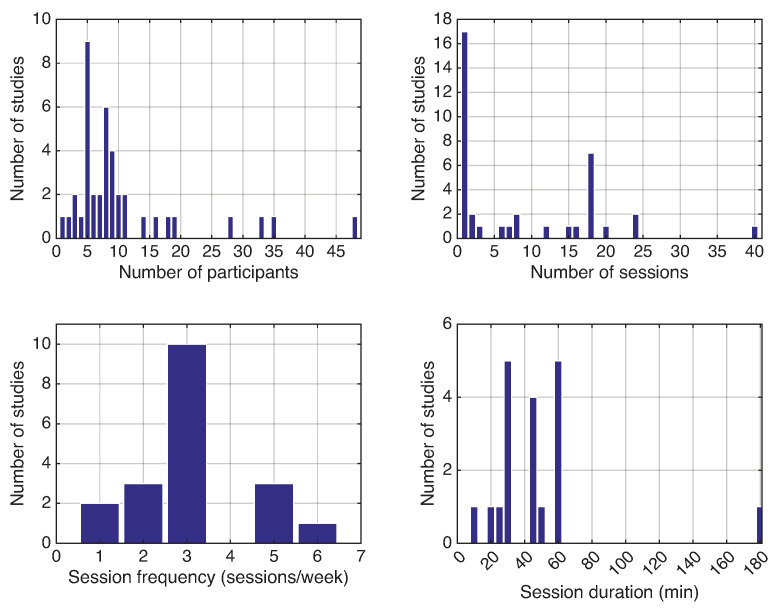
Overview of the experimental protocol characteristics. Bar plots display the number of studies versus number of participants, number of sessions, session frequency (sessions per week), and session duration (minutes) across the studies selected for research question 2. The total number of studies may vary between plots due to incomplete reporting of protocol parameters.

Figure 5 depicts an overview of the experimental protocol characteristics.

#### 3.4.4. Clinical Metrics

There is low homogeneity in the assessment of participants’ initial functional level and in the evaluation of the performance of the gravity compensation strategy. The Fugl-Meyer Assessment and shoulder and/or elbow range-of-motion tests are the only measures used across multiple studies, whereas the remaining assessments appear in five or fewer studies.

## 4. Discussion

This systematic review synthesized evidence on robot-assisted gravity compensation (GC) strategies for upper limb motor rehabilitation following neurological disorders. Across the 60 included studies, GC emerged as a fundamental but not thoroughly described component of rehabilitation robotics. While most robotic platforms integrate some form of weight-support mechanism, there is significant variability in how the compensatory torque is modeled, controlled, and reported. These findings complement prior reviews on rehabilitation robotics [7,88], which have primarily examined robotic systems from a broad perspective, focusing on overall device categories, assistive control strategies, and general clinical outcomes. More specifically, reviews such as [89] provide a comprehensive overview of computational and control approaches in neurorehabilitation robotics, while [9] summarizes recent advances in upper limb robotic systems and their clinical applications. In these works, GC is addressed as one of several assistive features and is not examined in isolation, with limited detail regarding its specific implementation, such as control strategies, modeling approaches, and parameter reporting.

### 4.1. (1) Which Robot-Assisted Gravity Compensation Strategies Have Been Used for Upper Limb Rehabilitation Following a Neurological Disorder?

Regarding the first research question, passive solutions, typically found in exoskeletons or suspension devices, rely on counterweights, springs, or elastic mechanisms to partially offset limb weight. Active GC is achieved through actuators and control algorithms that estimate gravitational torque based on simplified or anthropometric arm models. Hybrid approaches combine both mechanical and software-based components to reduce motor size requirements and enhance safety. Although adaptive, model-based GC schemes are increasingly implemented and can adjust assistance according to joint configuration or muscle effort [17,22], the lack of standardized terminology and detailed parameter reporting limits reproducibility and comparison across studies. Notably, only one study analyzed the stability of the GC control strategy. Only a few explicitly described key control features, such as the specific type of controller used or the frequency of the control loop, and none reported how the controller was tuned. Additionally, only two studies evaluated the device’s performance both statically and dynamically, and none reported the speed of movement in the dynamic tests. Similar concerns about heterogeneity in study designs and intervention characteristics have been raised in broader robotic rehabilitation research [9,89]. Table 5 presents a summary of the technical parameters reported.

### 4.2. (2) What Is the Current Clinical Evidence on the Effectiveness of These Strategies in Improving Motor Function?

Addressing the second research question, the review found consistent reports of improved arm movement and range of motion following GC-assisted training, particularly in individuals with severe impairments. Functional improvements measured by the Fugl-Meyer Assessment and kinematic indices were common, although gains were typically modest and not superior to other robotic or conventional therapies [15,18]. Among the randomized controlled trials, Fugl-Meyer Assessment scores improved in both intervention and control groups, with mean changes ranging from approximately 2.2 to 10.1 points in control groups and 3.3 to 8.0 points in intervention groups. These results mirror recent meta-analyses of robotic interventions, indicating small-to-moderate benefits for upper limb function [8,90].

The modest and non-superior clinical effects observed with GC-assisted interventions may be explained by several factors. First, GC primarily acts as an enabling mechanism that reduces the effort required to perform movements, rather than directly targeting task-specific motor recovery, which may limit its impact on functional outcomes. Second, substantial variability in training protocols, including differences in intensity, duration, and task specificity, may influence the extent to which GC contributes to meaningful improvements. Third, heterogeneity in participant characteristics, such as severity of impairment and stage of recovery, further complicates the interpretation of results and may mask differential effects across subgroups. Finally, commonly used outcome measures, such as the Fugl-Meyer Assessment, may not fully capture functional or task-level improvements facilitated by GC, particularly those related to movement quality or compensatory strategies. Together, these factors may help explain the modest clinical benefits reported across studies.

However, the strength of this evidence is limited by methodological quality. All randomized controlled trials showed some concerns for risk of bias, while all non-randomized clinical trials were judged at serious risk of bias, primarily due to confounding and concurrent interventions. As a result, it remains unclear to what extent the reported improvements can be attributed specifically to gravity compensation rather than to the broader rehabilitation context.

Evidence directly comparing GC strategies therefore remains insufficient; few studies isolate the contribution of GC from other assistive features. In addition, substantial heterogeneity in intervention protocols and participant characteristics further limits cross-study comparability. Consequently, the current evidence does not allow firm conclusions regarding whether different GC strategies yield meaningful differences in rehabilitation outcomes, either within or across different types of robotic systems.

### 4.3. Methodological Limitations

Several methodological limitations were identified. Study samples were generally small and heterogeneous in acuity, baseline motor ability, and outcome metrics. Training protocols ranged from single sessions to multi-week interventions, often lacking follow-up assessments. Reporting of GC implementation was inconsistent: key parameters such as the actual proportion of weight support, controller algorithms, and tuning were frequently omitted. These issues echo long-standing concerns about the transparency and standardization of robotic-rehabilitation research [7,89]. Moreover, the predominance of feasibility and pilot studies limits the generalizability of clinical conclusions.

### 4.4. Implications for Research and Practice

Clinically, gravity compensation appears most valuable as an enabling mechanism for individuals with moderate-to-severe upper limb weakness, allowing earlier initiation of task-oriented practice and increasing the feasible workspace. However, the reviewed evidence indicates that the benefits of GC are not determined by model fidelity or control complexity alone, as no strategy, whether passive, active, or adaptive, has demonstrated clear functional superiority over each other.

From a design perspective, the consistent lack of reporting on controller algorithms, tuning procedures, loop frequencies, and stability analyses highlight that transparency and reproducibility, rather than increased model complexity, are currently the main bottlenecks in comparing GC strategies. Without standardized reporting and validation, increasingly sophisticated strategies cannot be meaningfully compared, replicated, or translated into clinical practice. Future systems should therefore prioritize clear parameterization, open documentation, and task-level validation.

Finally, to assess and systematically compare GC strategies, large clinical trials must be accompanied by: (i) standardized technical reporting; (ii) outcome measures that explicitly link GC parameters to functional task performance; and (iii) adaptive control schemes that are evaluated for their clinical relevance, not only their engineering performance.

## 5. Conclusions

Robot-assisted gravity compensation is a fundamental yet still under-characterized component of upper limb rehabilitation robotics. The available evidence supports its feasibility and short-term benefits for improving movement quality, particularly in individuals with severe motor impairments. However, substantial variability in device design, incomplete technical reporting, small sample sizes, and methodological heterogeneity across studies currently prevent firm conclusions regarding the comparative effectiveness of different GC strategies. Although adaptive and model-based approaches are theoretically promising, they have not yet demonstrated clear functional superiority in controlled clinical settings. Establishing standardized technical reporting practices and systematically integrating adaptive, patient-specific gravity compensation into future systems will be essential to advance both technological development and clinical translation.

## Figures and Tables

**Table 1:** Overview of the studies included for clinical comparison that tested the devices in participants affected by neurological conditions other than stroke.

Reference	Neurological Disorder	Severity	Robot	Robot Classification	Study Design
Pedrocchi et al. [55]	SCI/MS	11 < FMA < 41	MUNDUS	Exoskeleton/ Active	Exploratory study
Fulton et al. [56]	SCI	N/A	SaeboMAS	End-effector/ Passive	Single-case report
Lebrasseur et al. [57]	MS/SMA	N/A	Gowing	End-effector/ Active	Exploratory study
Bastiaens et al. [42]	MS	EDSS $\bar{x}$ = 7.85	HapticMaster + FOCAL	End-effector/ Active + Sling-suspension/ Passive	Exploratory study
Keller et al. [58]	CP	MACS I-III	ArmeoSpring (pediatric)	Exoskeleton/ Passive	Clinical trial
Urendes et al. [59]	CP/SMA	MACS I-II	No name	Exoskeleton/ Passive	Pilot study

FMA = ‘Fugl-Meyer Assessment’; EDSS = ‘Expanded Disability Status Scale’; MACS = ‘Manual Ability Classification System’.

**Table 2:** Overview of the clinical trial studies included for clinical comparison.

	Robot	n *	Intervention	Control Condition	Major Findings
[60] ^1^	ArmeoSpring	48	15 sessions; 5/week, 1, 2, and 3D movements	Within/between groups; 3 groups classified by impairment level	The 3 groups showed significant FMA improvement; the group with highest level of impairment benefited more
[61] ^1^	ArmeoPower	35	40 sessions; 5/week	Within-subjects	Significant FMA, elbow flexion/extension and shoulder ROM improvements
[58] ^2^	ArmeoSpring (pediatric)	11	3 sessions playing videogames; 1/day	Within-subjects	Box and Block test improved from 0.45 to 3.96; kinematic assessments and Melbourne items did not change
[18] ^3^	ArmeoSpring	16	12 sessions per day; 6 days/week, over 2 weeks	Equal duration conventional therapy	Only robot group improved smoothness and shoulder adduction/abduction
[15] ^3^	T-WREX	14	23 sessions gradually reducing support	Equal duration conventional therapy	Robot group showed better FMA retention after 6 months and higher user preference
[62] ^3^	ArmeoBoom	33	18 sessions of reaching training; 3/week	Equal duration conventional therapy	Both groups improved FMA and reach distance equally but robot group reported higher motivation and enjoyment

GC = ‘gravity compensation’; FMA = ‘Fugl-Meyer Assessment’; ^1^ clinical trial and participants with stroke; ^2^ clinical trial and participants with cerebral palsy; ^3^ randomized clinical trial and participants with stroke; * study participants sample size.

**Table 3:** RoB 2 assessment of the three RCT studies.

	D1	D2	D3	D4	D5	Overall
Prange et al. [62]	Low	Some	Low	Low	Some	Some concerns
Housman et al. [15]	Some	Some	Some	Low	Some	Some concerns
Bartolo et al. [18]	Some	Some	Low	Low	Some	Some concerns

D1: bias arising from the randomization process; D2: bias due to deviations from intended intervention; D3: bias due to missing outcome data; D4: bias in measurement of the outcome; D5: bias in selection of the reported results.

**Table 4:** ROBINS-I assessment of the three non-RCTs studies.

	D1	D2	D3	D4	D5	D6	D7	Overall
Keller et al. [58]	Serious	Moderate	Low	Moderate	Serious	Moderate	Moderate	Serious
Chan et al. [60]	Serious	Moderate	Low	Moderate	Moderate	Moderate	Moderate	Serious
Calabro et al. [61]	Serious	Moderate	Low	Moderate	Moderate	Moderate	Moderate	Serious

D1: bias due to confounding; D2: bias due to selection of participants; D3: bias in classification of interventions; D4: bias due to deviations from intended interventions; D5: bias due to missing data; D6: bias in measurement of outcomes; D7: bias in selection of the reported results.

**Table 5:** Summary of technical parameters reported of the active gravity compensation strategies.

	Robot Classification	Model of the Arm		Controller	Control Details Reported	System Evaluation
		DoF	Dimensions			
[38]	Exoskeleton	4	Anthropometric data	Torque-Feedforward	None	Static + dynamic
[17]	Exoskeleton	NR	Anthropometric data	Torque-Feedforward	Partial	Dynamic
[30]	Exoskeleton	4	Anthropometric data	Torque-Feedforward	Partial	Dynamic
[51]	Exoskeleton	5	Anthropometric data	Torque-Feedforward	None	Static
[21]	Exoskeleton	5	Anthropometric + empirical data	Torque-Feedforward	None	Static + dynamic
[22]	End-effector	2	Anthropometric data	Force-Feedback	Partial	Static
[52]	End-effector	4	Anthropometric data	Torque-Feedforward	Partial	Dynamic
[16]	End-effector	4	NR	NC	None	Static
[49]	End-effector	4	Empirical data	Torque-Feedforward	None	None
[19]	End-effector	2	Anthropometric data	Force-Feedforward	Partial	None
[40]	Sling-suspension	2	NR	Torque-NC	None	Dynamic
[53]	Sling-suspension	2	Empirical data	NC	None	None

Control details considered: stability analysis, tuning of the controller, frequency of the control loop; DoF = ‘degrees of freedom’; NR = ‘not reported’; NC = ‘not clear’.

## Data Availability

All data supporting the findings of this study are included within the article or cited in the referenced materials.

## Abbreviations

The following abbreviations are used in this manuscript:

CP	Cerebral palsy
CNS	Central Nervous System
DoF	Degrees of freedom
GC	Gravity compensation
MS	Multiple Sclerosis
NC	Not clear
NR	Not reported
RoB 2	Cochrane risk of bias tool
ROBINS-I	Risk Of Bias In Non-randomized Studies—of Interventions
SCI	Spinal Cord Injury
